
JOURNAL INFORMATION
==============================
NLM Title Abbreviation: ScientificWorldJournal
Journal ID: ScientificWorldJournal
NLM Journal ID: 101131163
Journal ID: TSWJ

The Scientific World Journal

ISSN: 2356-6140
EISSN: 1537-744X
Publisher: Wiley

ARTICLE INFORMATION
==============================
PMCID: PMC6084515
PMID: 30147510
DOI: 10.1100/tsw.2001.179
Article ID: 517073
Article version: 1
Subjects: Short Report

Morphological and Biochemical Characterization of a Cell Death Process Induced by E3, a New Synthetic Diarylsulfonilurea Analogue

Alonso M. 1 *
Miglaccio M. 1
Encío I. 1
Asumendi A. 3
Martinez-Merino V. 2
Hilario E. 3
García M. 3
1 Department of Health Sciencies, Public University of Navarra, Spain
2 Department of Applied Chemistry, Public University of Navarra, Spain
3 Department of Cellular Biology, University of the Basque Country, Spain
*M. Alonso: gcbalrom@lg.ehu.es
Publication date: 2001
Electronic publication date: 2001 Dec 1
Volume: 1
First page: 50
Last page: 50
Copyright: Copyright © 2001 M. Alonso et al.
Copyright year: 2001
License: This is an open access article distributed under the Creative Commons Attribution License, which permits unrestricted use, distribution, and reproduction in any medium, provided the original work is properly cited.
License URL: https://creativecommons.org/licenses/by/3.0/

==============================
Miami *Nature Biotechnology* Short Reports
*TheScientificWorld* (2001) 1(S3), 50SR
ISSN 1532-2246; DOI 10.1100/tsw.2001.179

### MORPHOLOGICAL AND BIOCHEMICAL CHARACTERIZATION OF A CELL DEATH PROCESS INDUCED BY E3, A NEW SYNTHETIC DIARYLSULFONILUREA ANALOGUE

M. Alonso* (1), M. Miglaccio (1), I. Encío (1), A. Asumendi (3), V. Martinez-Merino (2), E. Hilario (3) and M. García (3)
(1)Department of Health Sciencies, Public University of Navarra, Spain. (2) Department of Applied Chemistry, Public University of Navarra, Spain. (3) Department of Cellular Biology, University of the Basque Country, Spain.
* gcbalrom@lg.ehu.es

**INTRODUCTION.** With a view to finding new compounds with improved antitumoral activity and reduced secondary effects, our group, using theoretical structure-activity studies, has designed several analogues and derivatives of diarylsulfonilureas (1). These compounds have been synthesized and tested *"in vitro"* on a panel of human tumor cell lines and showed an antitumoral activity (2). In the present work, we have characterized the mechanisms involved in the cell death process induced by one of these products called E3 in a CCRF-CEM cell line.

**METHOD.** The lymphoblastic cell line, CCRF-CEM, was routinely grown in RPMI 1640 supplemented with 1 mg/ml gentamicine, 2 mM glutamine and 10% fetal calf serum. The morphological and ultrastructural analyses were performed using semithin sections and transmission electron microscopy. Cytometry was employed to monitor the cell cycle, mitochondria activity and cell death using appropriated fluorochromes. Western Blot using specific antibodies was performed to study the expression of apoptosis-related proteins. DNA fragmentation in internucleosomic ladders was analyzed by agarose gel electrophoresis. We also determined the level of lactate dehydrogenase released into the culture medium as an indicator of necrosis and employed the N-acetyl-1-cysteine (3) to counteract the possible effect of reactive oxygen intermediates.

**RESULTS.** We have observed that cell death induced by E3 in the CCRF-CEM line is dose-dependent. Cell death analysis, evaluated with transmission electron microscopy confirmed that some cells displayed the initial morphological signs of apoptosis, namely highly condensed chromatin around the nuclear membrane and fragmentation of the nucleus into small rounded chromatin bodies.

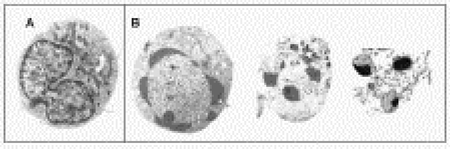

**Fig. 1.** Morphological characteristics (A) of typical untreated CCRF-CEM cells. (B) E3 treated CCRF-CEM cells.

The mitochondrial activity is diminished gradually as shown by progressive change in the level of rhodamine 123 uptake. The decrease in mitochondrial activity was accompanied by an increased release of lactate dehydrogenase into the culture medium. Moreover, we observed that N-acetyl-1-cysteine completely blocked the antiproliferative and apoptotic-inducing effects of E3, suggesting that an oxidative mechanism may be involved. Additionally some apoptosis related proteins are not modified during this cell death process, suggesting an abortive apoptotic process followed by a necrotic destruction.

**DISCUSSION.** In the light of the results obtained we considered that E3, a new synthetic diarylsulfonilurea analogue, elicits its action via mitochondrial alteration with hyperproduction of reactive oxygen species and initiation of apoptogenic pathways. Since, the mitochondrial dysfunction entails a bioenergetic alteration associated with ROS (Reactive Oxygen Species) production culminating in the disruption of plasma membrane integrity in a secondary necrotic process.

**REFERENCES.**

1. Grindey, G.B. (1988) Proc. Am. Assoc. Cancer Res. 29, 535
2. Arteaga, C. (2000) Antitumoral activity of new analogues of DSU, Doctoral Thesis, The Public University of Navarra
3. Buttke, T.M. and Sandstrom, P.A. (1994) Inmunol.Today 15, 7-10
